# The role of family meal frequency in common mental disorders in children and adolescents over eight months of follow-up

**DOI:** 10.1371/journal.pone.0243793

**Published:** 2021-02-04

**Authors:** Beatriz Tosé Agathão, Diana Barbosa Cunha, Rosely Sichieri, Claudia Souza Lopes

**Affiliations:** Department of Epidemiology, Institute of Social Medicine, State University of Rio de Janeiro (UERJ), Rio de Janeiro, Brazil; Shahjalal University of Science and Technology, BANGLADESH

## Abstract

**Objective:**

This study evaluated the influence of family meal frequency on the occurrence of common mental disorders (CMD) in children and adolescents over eight months of follow-up.

**Design:**

Data from this longitudinal study were derived from the PAAPAS—*Parents*, *Students*, *Community Health Agents*, *and Teachers for Healthy Eating—*community trial. CMD were evaluated using the General Health Questionnaire. Frequency of family meals (breakfast and dinner) was categorized as “breakfast and dinner regularly with the family”, “at least breakfast or dinner regularly with the family”, and “does not have any meal regularly with the family.” The effect of family meal frequency on CMD was analyzed using generalized estimation equations with log-binomial models for repeated measures.

**Setting:**

This study was conducted in public schools (*N* = 18) of Duque de Caxias, Rio de Janeiro, Brazil, in 2016.

**Participants:**

Children (aged 9–11 years) and adolescents (aged 12–17 years) from the fifth and sixth grades (*N* = 2,743).

**Results:**

These findings suggested that regular family meals were a protective factor for mental health. The adjusted relative risk of CMD was 0.75 (95% confidence interval = 0.69–0.83) for those who had two family meals regularly and 0.87 (95% confidence interval = 0.77–0.97) for those who had only one regular family meal, compared to students who had no regular family meals.

**Conclusions:**

Potential strategies that educate and encourage families about the mental health benefits of eating regular meals together must be explored and implemented.

## Introduction

Mental disorders mainly include non-psychotic mental disorders (90%) [1], such as mood, anxiety, and substance use disorders [2]. Given the high prevalence of such conditions in the general population, they are therefore called *common mental disorders (CMD)* [2–5]. Findings pooled from 174 surveys across 63 countries indicate that, on average, 17.6% of adults experienced a common mental disorder within the past 12 months and 29.2% across their lifetime [2]. CMD affect different age groups and are characterized by the presence of depressive and anxious symptoms. Furthermore, somatic and nonspecific complaints, such as fatigue, forgetfulness, irritability, concentration, and sleep difficulties, are also common features of CMD [6].

Problems related to mental health in childhood and adolescence constitute an important part of the global burden of diseases (10%–20%) [7]. A meta-analysis was performed to summarize the prevalence of CMD in adolescents worldwide (10 to 19 years old) and a total of 43 studies were included. The global prevalence found was 25%–31%, depending on the cutoff point used [8]. In Brazil, some studies have been devoted to assessing the prevalence of CMD in this age group, finding a range of about 20–30% [4, 9–11]. At the end of childhood and adolescence, which are vulnerable periods involving important biological and social changes, CMD might affect academic performance, affective relationships, and potential initial traits of more severe mental disorders [4]. Some risk factors for CMD in children and adolescents described over time include the absence of a structured home, violence, early maternity/paternity, and conditions of extreme poverty [2, 12, 13].

According to the literature, several aspects of the family environment influence the healthy development of children and adolescents. Among them, family meal patterns have been studied to identify their impact on the nutritional [14, 15] and, more recently, mental health [16, 17] of children and adolescents. Concurrently, mealtime routines as daily traditions have changed significantly in the past decade, with a progressive decline in frequency associated with a social deconstruction of a model comprising three meals a day as well as the choices for “*fast food”* [18, 19].

A recent systematic review of the effects of family meals on the psychosocial aspects of children and adolescents identified that a frequency of at least five times a week widely denotes a regular practice of family meals and that regularity decreases as the individual approaches adulthood [16]. Other factors, including geographic location and cultural issues, also showed marked differences in the frequency with which the family participated in meals, although the selected studies only included medium- and high-income countries [16]. Furthermore, the seven identified longitudinal studies focused on eating disorders and/or externalizing symptoms (e.g., risk behaviors, such as substance use and violent behavior). Of these, only three [20–22] focused on depressive symptoms, self-esteem perception, and body image as an outcome.

Evidence suggests that regular family meals can positively affect the self-esteem, well-being, and school performance of children and adolescents [21, 23, 24]. Additionally, some studies showed an inverse association of this family routine with risk behaviors, such as alcohol and drug use, eating disorders, and depressive symptoms [16, 22, 25]. In young children, the presence of parents at mealtimes facilitates language development, communication skills, and school involvement, thus reducing the risk of problem behaviors and conduct disorders [26].

Given the importance of CMD in children and adolescents, the global burden and long-term implications associated with such disorders, investigating factors that may alter the course of this condition merit attention. Therefore, evaluating the influence of family meals on the mental health of young people in low-income areas may support public strategies to encourage modifiable patterns within families. This study evaluated the influence of family meal frequency on the occurrence of CMD in schoolchildren and adolescents over eight months of follow-up.

## Materials and methods

**Fig 1:**
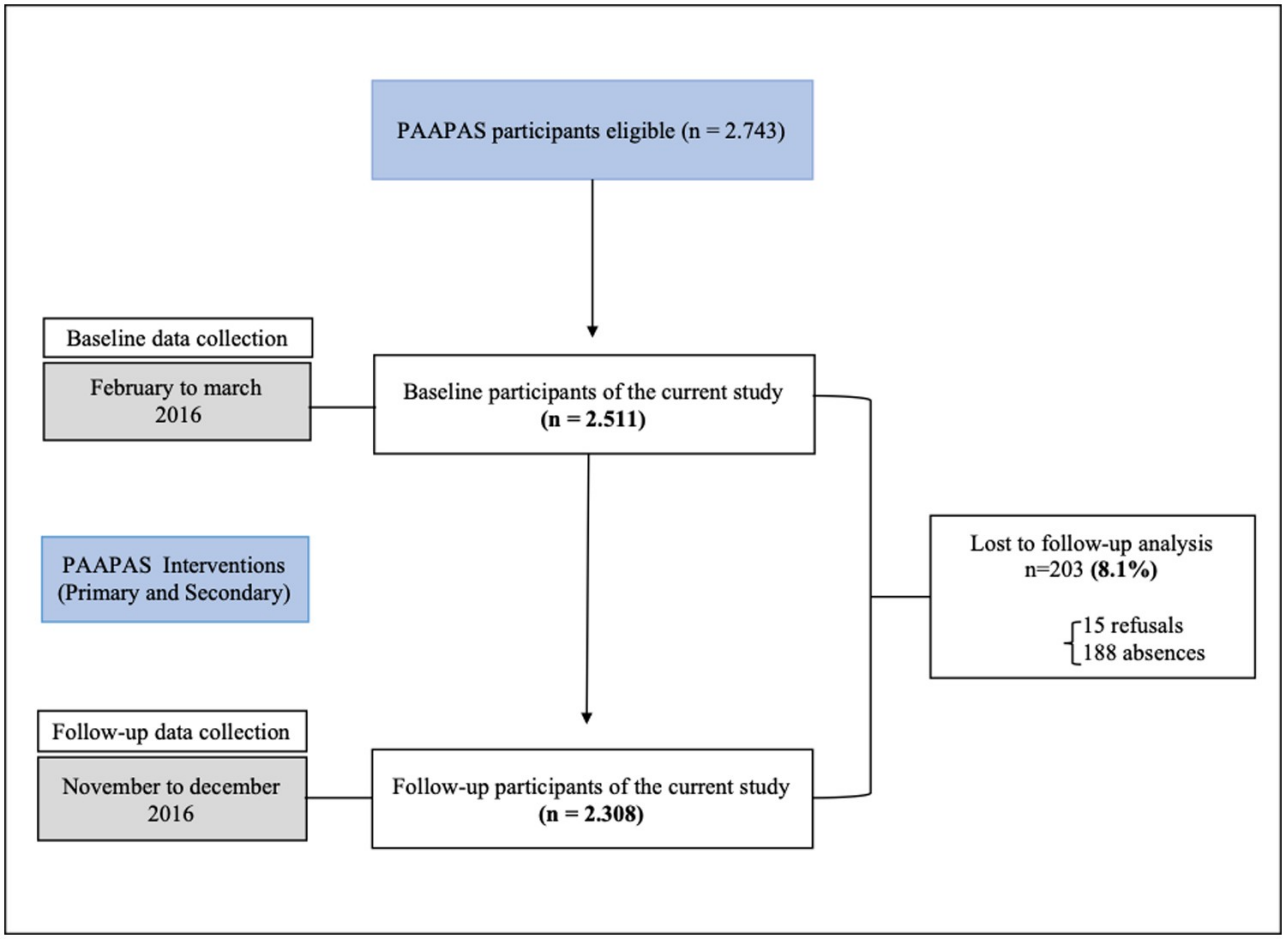
Flow diagram of the current study with data from PAAPAS 2016.

Data from this prospective study (Fig 1) were derived from the PAAPAS (Parents, Students, Community Health Agents, and Teachers for Healthy Eating) project (registration number NCT02711488), a randomized community-controlled trial conducted in 2016 among students from public schools in the city of Duque de Caxias, Rio de Janeiro, Brazil. For PAAPAS study, two of four districts in Duque de Caxias were included, and from 45 municipal schools, 18 schools with fifth and sixth grades classes were selected. All students enrolled in the 5th and 6th grades of selected schools were considered eligible for the study. Schools were randomized half in the control group and half in the intervention group based on number of students using opaque envelopes, in the presence of investigators not involved in the study (1,406 students were allocated to the intervention group and 1,337 to the control group).

Duque de Caxias (population: 842,686), located in the metropolitan area of Rio de Janeiro and 27 km from the state capital [27], is also one of the poorest areas in Rio de Janeiro. This study included two of the four districts in Duque de Caxias, and eighteen of the forty-five municipal schools with fifth and sixth grades were selected.

The PAAPAS proposed to reduce excessive weight gain among children and adolescents by combining primary intervention at schools with secondary intervention at home through trained community health agents (CHA). Primary intervention in the school—conducted by trained teachers—provided the overall basis for a healthy lifestyle, with activities focused on encouraging students to improve the consumption of healthy foods and encouraging them to increase physical activity and reduce sedentary behavior; these included strategies such as culinary classes and group discussions and games about fruits and the amount of sugar in different drinks culinary classes. For adolescents diagnosed with excessive weight, guidance at home was provided by CHA as an additional motivation to change these behaviors.

The sample size of PAAPAS was calculated considering a power of 80% and a significance level of 5%, assuming a prevalence of overweight/obesity of 20% (which was the main outcome of the background study). This calculation resulted in a necessary sample size of 2,340 participants. Comprehensive descriptions of each intervention, sample size, study design, and randomized procedures can be accessed in the PAAPAS [28]. The data were collected in two phases: at baseline (March and April 2016) and again after primary and secondary interventions (November and December 2016).

The protocol was approved by the Ethics Committee of the Institute of Social Medicine (Comitê de Ética do Instituto de Medicina Social—CAAE: 10471313.2.0000.5260). Written informed consent was obtained from all participants’ parents. Students with physical or mental disabilities or who were pregnant or lactating were considered ineligible and were excluded from the study.

### Measures

Anthropometric measurements and a questionnaire were administered at the beginning (pre-intervention) and end (post-intervention) of the school year. Students completed a structured questionnaire using personal digital assistants (PDAs) under the supervision of field researchers. Before each data collection, the supervisors explained the procedure for completing the digital questionnaire and addressed the students’ doubts throughout the process. Upon completion, the same group conducted a conference check on the PDAs to detect device errors.

Information on the outcome measure was obtained from the short version of the General Health Questionnaire (GHQ-12) [29], applied at both baseline and follow-up. It is a 12-item self-report instrument used to assess non-psychotic mental disorders and was developed to identify symptoms of depression and anxiety, inability to deal with ordinary situations, and lack of self-confidence, which are commonly found in the general population. Each GHQ item has four response options and a reference period of two weeks before completing the questionnaire. The questionnaire has been validated for the Brazilian population using a structured psychiatric interview as the gold standard and has been found to have good psychometric properties [30]. The Brazilian version of the GHQ at the cutoff point of 4/5 showed a sensitivity of 76% and specificity of 82% [31]. Each item was recorded as “absent” or “present” (0 or 1, respectively) and then summed. A cutoff point of at least 5 positive responses in 12 items used by Fortes [32] to detect severe-intensity mental disorders was applied.

The frequency of family meals (FFM) was assessed using two baseline questions: “How many times in a week do you have breakfast with a parent or guardian?” and “How many times in a week do you have dinner (including sandwiches, omelets, and other food rather than food plate) with a parent or guardian?” For each question, there were five response options: “never or almost never,” “one to two times a week,” “three to four times a week,” “five to six times a week,” and “every day.” First, the responses to the variables regarding breakfast and dinner with family were categorized into regular (five or more times in the week) and irregular (up to four times a week). Previous studies have utilized the same categorization [33, 34]. Afterward, responses on the variables (breakfast and dinner) were combined, comprising “breakfast and dinner regularly with the family”, “at least breakfast or dinner regularly with the family”, and “does not have any meal regularly with the family.” The nomenclature used in the tables for the categories of the exposure variables mentioned above were: “All meals,” “One meal,” and “None,” respectively.

According to the Brazilian Economic Classification Criteria [35], the socioeconomic status of the participants’ families was determined from information on 11 durable goods at home (car, motorcycle, computer, refrigerator, freezer, washing machine, dishwasher, microwave, and DVD player) and characteristics of the residential area, such as piped water and paved streets. An indicator was elaborated to represent the socioeconomic position of the families, based on a study by Barros and Victora [36]. The first component generated by principal component analysis (PCA), which captures the largest possible amount of data variability with a single linear combination, was retained [37, 38]. Therefore, the durable goods at home and residence characteristics were used in a 1-factor PCA model, which obtained an eigenvalue of 2.05 and a Cronbach’s alpha of 0.21 and explained 19.3% of the total variance. Subsequently, the factorial score was divided into quintiles.

### Statistical analyses

Descriptive analyses at the baseline and follow-up were estimated: means and standard deviations for continuous variables, and proportions for categorical variables. To test the differences between the proportions of the study variables and the frequency of CMD, chi-square tests were applied. Linear regressions were conducted to test the trend between the socioeconomic position and CMD. All tests performed considered a significance level of 5%. Descriptive analyses were performed using the Stata software, version 13.

To evaluate the influence of family meal frequency on CMD, longitudinal analyses were applied using generalized estimating equations (GEE), which extends the generalized linear model to handle longitudinal data by maximum likelihood estimation of the parameter vector β, with log-binomial models for repeated measures. For this, we used the *GENMOD* procedure from the Statistical Analysis System (SAS) version 9.4, with the independent type structure of the working correlation, proceeded by the estimation of relative risks and 95% confidence intervals.

Initially, the model included “time,” “FFM,” and interaction term “time*FFM,” which allowed the evaluation of potential differences between exposure groups in response change over time. Although no specific interventions for family meal frequency and CMD were performed, the group variable was inserted into the adjustment to eliminate residual confounding, thus the models were adjusted for sex, age, asset indicator, and group but were not statistically significant for the interaction term. Stratified analyses by sex and age group also revealed no statistical significance.

Considering that the longitudinal analysis showed that the relationship between family meal frequency and CMD does not vary over time, we withdrew the interaction term and conducted a multivariate model including the time variable and adjusted for the previously mentioned confounding variables.

## Results

As shown in Fig 1, of the 2,743 eligible school students, 2,528 participated in the baseline. About 2,511 (99.3%) students answered GHQ-12 in the first phase of the study, while 2,308 responded in the follow-up; therefore, 8.1% of the sample was lost between the two data collections. Follow-up losses were mainly due to participants’ refusals (15 students) and absences.

The proportions of boys (52%) and girls and children (56.2%) and adolescents were close. Regarding the frequency of family meals, half of the students regularly had breakfast and dinners with family (at least five times a week) and 25.4% had no regular meals with the family. Almost 24% of the students had either breakfast or dinner with their families five or more times a week. In general, the sample studied had an average CMD score of 11.7 (Table 1).

**Table 1:** Characteristics of the sample at baseline.

Variables	Total n (%)
**Sex**	
Boys	1.427 (52.0)
Girls	1.316 (48.0)
**Age (years)**	
9 to 11	1.421 (56.2)
12 to 17	1.107 (43.8)
**Frequency of Family meals (Breakfast / Dinner)**	
Two meals^1^ (%)	1.277 (50.8)
One meal^2^ (%)	600 (23.8)
None^3^ (%)	639 (25.4)
**Common mental disorders frequency**	
Yes	834 (33.2)
No	1.678 (66.8)
**Common mental disorders score**	**Mean (SD**^+^**)**
0–36	11.7 (8.1)

^1^Two meals—Breakfast and dinner regularly with the family.

^2^One meal—At least breakfast or dinner regularly with the family.

^3^None—Does not have any meal regularly with the family.

^+^Standard deviation.

The frequency of CMD was 33.2% at baseline (boys = 36.9%; girls = 29.2%) and 32.2% at follow-up (boys = 35.1%; girls = 29%). The presence of CMD was more frequent among adolescents than in children in both phases of the study and increased with socioeconomic status at baseline and follow-up. At baseline and follow-up, it was observed that as the frequency of family meals decreased, the frequency of CMD increased, with almost 42% and 38.1% of those who had no regular family meals at baseline and follow-up, respectively, showing signs of CMD (Table 2).

**Table 2:** Frequency of common mental disorders (CMD) at baseline and follow-up according to population characteristics.

Variables	Common mental disorders			
	Baseline (%)		Follow-up (%)	
	n = 834 (33.2)		n = 742 (32.2)	
**Sex**		***p* value**^+^		***p* value**^+^
Boys	486 (36.9)	<0.001	418 (35.1)	0.002
Girls	348 (29.2)		324 (29.0)	
**Age groups (years)**		***p* value**^+^		***p* value**^+^
9 to 11	447 (31.7)	0.06	380 (30.2)	0.017
12 to 17	387 (35.2)		295 (35.2)	
**Socioeconomic Position**		*p* value*		*p* value*
1° quintile (lower)	141 (27.9)	<0.001	104 (24.4)	<0.001
2° quintile	143 (28.7)		118 (28.9)	
3° quintile	156 (30.5)		133 (31.4)	
4° quintile	158 (32.1)		129 (31.5)	
5° quintile (higher)	235 (46.9)		185 (45.0)	
**Frequency of Family meals (Breakfast / Dinner)**		***p* value**^++^		***p* value**^++^
Two meals (%)	374 (29.4)	<0.001	310 (29.1)	<0.001
One meal (%)	193 (32.2)	<0.001	167 (32.9)	0.082
None (%)	267 (41.8)	Ref	195 (38.1)	Ref

^+^*p* value of chi-square test

*p trend of linear regression test

^++^Chi-square test between “all meals and no meal” and “at least one meal and no meal,” and common mental disorders.

Table 3 presents the effect of family meal frequency on common mental disorders over time. Regularly eating breakfast and dinner with family protects against the occurrence of a CMD in both unadjusted and adjusted models. The crude relative risk of CMD was 0.73 (95% confidence interval = 0.66–0.81) for those who had two family meals regularly and 0.81 (95% confidence interval = 0.72–0.91) for those who had only one regular family meal, compared to students who had no regular family meals. These relationships were attenuated but remained significant even after adjustment for gender, age, socioeconomic status, and group to which they belonged in the background study (intervention or control), with an adjusted RR 0.75 (95% confidence interval = 0.69–0.83) and adjusted RR 0.87 (95% confidence interval = 0.77–0.97), respectively.

**Table 3:** Regression coefficients (β), standard deviation (SD), and relative risks (RR) of CMD, according to time of follow-up and frequency of family meals (FFM).

Variables		Model without adjustment for baseline measures			Adjusted model for baseline measures*		
		β^+^	SD^+^	RR^+^ (95% CI)	β	SD	RR (95% CI)
**Time**		0.0306	0.0378	0.96 (0.90–1.04)	0.0263	0.0377	0.97 (0.90–1.05)
	**Two meals**	-0.3172	0.0528	0.73 (0.66–0.81)^++^	-0.2875	0.0514	0.75 (0.69–0.83)^++^
**FFM**	**One meal**	-0.2124	0.0625	0.81 (0.72–0.91)^++^	-0.1438	0.0603	0.87 (0.77–0.97)^++^
	**None**			Reference			Reference

*Age, sex, assets indicator, and group.

^+^Regression coefficients (β), standard deviation (SD), and relative risks (RR).

^++^*p* <0.05.

## Discussion

This prospective study showed that a lack of family meals was associated with a higher frequency of common mental disorders, both at baseline and follow-up in a cohort of 2,511 school children and adolescents. Additionally, our central findings revealed that the impact of family meal frequency on CMD did not change but persisted over time. Moreover, a gradient effect was observed, since this protective factor was higher in those who ate both meals (breakfast and dinner) with family than in students who only had breakfast or dinner with family, with the same regularity. Finally, students who had regular family meals experienced a significant reduction in the risk of CMD compared to those who had no regular family meals.

Although no other studies with a specific interest in CMD have been identified, our results are consistent with most studies on the relationship between family meals and the mental health of children and adolescents [16, 17, 21, 23]. In a survey of 99,462 students in the sixth to twelfth grade from 213 cities across the United States, Fulkerson et al. [23] observed that adolescents who have dinner with their family five or more times per week were significantly less likely to be depressed compared to adolescents who reported eating one family dinner or less per week. Data from a school-based survey of 4,746 adolescents from diverse communities in Minneapolis and St. Paul in the U.S. showed similar significant findings, but only among girls. It was reported that a one-unit increase in family meal frequency was associated with reduced odds of high depressive symptoms after controlling for family connectedness and socio-demographic variables [21]. Recent data from a representative survey of the health and well-being of 8,500 school students in New Zealand showed that a greater frequency of family meals (≥5 times a week) was associated with fewer depressive symptoms and emotional difficulties and better well-being. These findings advance our knowledge about family meals and mental health since they demonstrate significant relationships between family meal frequency and positive dimensions of mental health, such as well-being, which is also important in the investigation of CMD [17]. However, the studies identified so far have no specific interest in CMD, thus gaps in the literature still exist.

Two recent reviews had similar conclusions about the impact of family meals on adolescent health. The first [39] study reviewed literature regarding the association between family meals and adolescent risk outcomes: drugs, aggressive and/or violent behavior, poor school performance, inappropriate sexual behavior, mental health problems, and eating disorders. Concerning mental health problems, it was found that family meal frequency was associated with a reduction in depressive symptoms and suicide in adolescents, indicating that regular family meals may protect young people’s mental health. The second [16], a systematic review of the effects of frequent family feeding on psychosocial outcomes in children and adolescents showed that frequent family meals were inversely associated with eating disorders, risk behaviors, and symptoms of depression or suicidal ideation. A positive relationship was found between frequent family meals and increased self-esteem and learning ability.

A range of arguments exists regarding the protective effect of family meals on children and adolescents’ mental health. Family meals represent a ritual for parents to emotionally connect with children through feelings of closeness and belonging [40, 41] and to identify early changes in existing patterns—such as changes in dress, friendships, and academic performance—that may indicate deviation in behavior [39]. Conflicts of interest and activities for school-age children (ages 6–12) and parents, demanding working hours, and economic difficulties create tensions in families that can negatively influence involvement in family occupation. Family meals are a family moment that offers the opportunity for families to connect despite the intense ongoing demands of modern life [42].

The strengths and limitations of this study should be considered. Among these strengths, we highlight the robustness of longitudinal analysis through a generalized estimating equation with log-binomial models for repeated measures, which provided support for the causal role of family meals on CMD. Furthermore, to the best of our knowledge, this study is the first to evaluate this relationship. The present study has certain limitations. Given that family meals may be a marker for other aspects of family structure, measures related to parental characteristics—such as parenting style, employment, education levels, and quality of family communication—must be analyzed to better understand these relationships. However, a study by Musick and Meiers [35] demonstrated that family dinners were linked to lower levels of depressive symptoms despite considering these family characteristics.

## Conclusion

Findings from this pioneering study suggest the importance of regular family meals as a protective factor for mental health. Mental disorders are among the challenges faced by health services. Traits of psychological distress, which can significantly affect development, are often found in children and adolescents before formal psychiatric diagnoses, thus early identification of CMD and its accompanying risk and protective factors is required. Potential strategies educating and encouraging families about the mental health benefits of eating regular meals together must be explored and implemented. Future research should examine the influence of other factors of the family structure, beyond family meal frequency.
